# Pyrrolylquinoxaline-2-One Derivative as a Potent Therapeutic Factor for Brain Trauma Rehabilitation

**DOI:** 10.3390/pharmaceutics12050414

**Published:** 2020-04-30

**Authors:** Elizaveta A. Dutysheva, Marina A. Mikeladze, Maria A. Trestsova, Nikolay D. Aksenov, Irina A. Utepova, Elena R. Mikhaylova, Roman V. Suezov, Valery N. Charushin, Oleg N. Chupakhin, Irina V. Guzhova, Boris A. Margulis, Vladimir F. Lazarev

**Affiliations:** 1 Institute of Cytology of the Russian Academy of Sciences, 194064 St. Petersburg, Russia; linza.uri@mail.ru (E.A.D.); marinamikeladze.cytspb@gmail.com (M.A.M.); aksenovn@gmail.com (N.D.A.); mikhailovaer@yandex.ru (E.R.M.); roman.suezov@gmail.com (R.V.S.); irina.guzh@gmail.com (I.V.G.); margulis@incras.ru (B.A.M.); 2 Ural Federal University, 620002 Ekaterinburg, Russia; mtrescova@mail.ru (M.A.T.); irina-utepova@yandex.ru (I.A.U.); charushin@ios.uran.ru (V.N.C.); chupakhin@ios.uran.ru (O.N.C.); 3 Postovsky Institute of Organic Synthesis, Ural Branch of the Russian Academy of Sciences, 620108 Ekaterinburg, Russia

**Keywords:** traumatic brain injury, cerebrospinal fluid, Hsp70, small molecule, apoptosis

## Abstract

Traumatic brain injury (TBI) often causes massive brain cell death accompanied by the accumulation of toxic factors in interstitial and cerebrospinal fluids. The persistence of the damaged brain area is not transient and may occur within days and weeks. Chaperone Hsp70 is known for its cytoprotective and antiapoptotic activity, and thus, a therapeutic approach based on chemically induced Hsp70 expression may become a promising approach to lower post-traumatic complications. To simulate the processes of secondary damage, we used an animal model of TBI and a cell model based on the cultivation of target cells in the presence of cerebrospinal fluid (CSF) from injured rats. Here we present a novel low molecular weight substance, PQ-29, which induces the synthesis of Hsp70 and empowers the resistance of rat C6 glioma cells to the cytotoxic effect of rat cerebrospinal fluid taken from rats subjected to TBI. In an animal model of TBI, PQ-29 elevated the Hsp70 level in brain cells and significantly slowed the process of the apoptosis in acceptor cells in response to cerebrospinal fluid action. The compound was also shown to rescue the motor function of traumatized rats, thus proving its potential application in rehabilitation therapy after TBI.

## 1. Introduction

The processes accompanying the recovery from traumatic brain injury (TBI) can cause additional damage to the brain, so called secondary damage [1]. Secondary damage factors include changes in blood supply to the brain, ischemia, cerebral hypoxia, inflammation, cerebral edema, and increased intracranial pressure [2]. The most common TBI effects that may occur over time due to secondary damage are memory deficit [3], motor dysfunction [4], and anxiety-like behavior [5].

At the cellular level, factors that induce secondary damage are the burst of reactive oxygen species, excessive release of the glutamate neurotransmitter, the influx of calcium and sodium ions into neurons, and mitochondrial dysfunction, which ultimately leads to the death of glial and neuronal cells, most often through apoptosis [6,7] or necrosis mechanisms [8]. The potentially toxic products of cell death can accumulate in the cerebrospinal fluid (CSF) as they are transported from the interstitial fluid through the glymphatic system [9]. Since the chemical composition of the CSF reflects that of the intercellular space, the fluid is often used to search for markers of neurodegenerative pathologies stemming from head injuries and as a convenient model for research [10]. As we showed previously, protein aggregates can be detected in the CSF of rats after TBI, and the CSF itself may have a cytotoxic effect imitating post-trauma conditions in the animal brain [11].

An important role in protecting cells from pathogenic processes is played by molecular chaperones, in particular, heat shock protein Hsp70 (HSPA1A), which recognizes and degrades polypeptides with impaired conformation and is involved in blocking a variety of apoptosis pathways [12,13]. The expression of hsp70 is regulated by the Hsf1 transcription factor; the active Hsf1 active phosphorylated trimer specifically binds to the heat shock element (HSE) on the *hsp70* promotor region, which triggers gene transcription.

Hsp70 protein prevents the formation of so-called apoptosomes, binds the apoptosis-inducing factor AIF, and prevents cell death by inhibiting procaspase-3/-7 activation [14,15]. Another mechanism of the Hsp70 chaperone action is binding mutant, improperly folded proteins, and inhibiting their aggregation [16,17]. Importantly, compounds that activate the synthesis of Hsp70 have a therapeutic effect in numerous models of neurodegeneration, including Parkinson’s disease [18], Alzheimer’s disease [19], spinocerebellar ataxia type 14 [20], spinal and bulbar muscular atrophy [21], and others. In regards to the potential role of Hsp70 in the post-trauma recovery period, it was found that the knockout of the chaperone gene in mice with an experimental head injury led to a significant increase in the lesion zone [22], and therapy using the Hsp70 17-AAG inducer reduced bleeding in injured mice [23]. Additionally, the treatment of traumatized rats with propolis that induced Hsp70 synthesis led to a reduction of the apoptosis level in the rat’s brain [24]. Another activator of Hsp70 synthesis, celastrol, was found to protect neural cells from reactive oxygen species, a potent inducer of secondary damage [25]. In conclusion, these data show that the chaperone may play a therapeutic role in the curation of the trauma-associated pathology similar to what it does in other neurodegenerative pathologies.

The purpose of this work was to test a novel inducer of Hsp70 chaperone synthesis as a potential drug for rehabilitation therapy after TBI. We screened compounds collection of pyrrolyl- and indolylazines, discovered a powerful inducer of Hsp70 synthesis, and tested it in models of post-traumatic recovery.

## 2. Materials and Methods

### 2.1. Reporter System and Screening

For the searching of compounds that activate the synthesis of Hsp70, more than 50 heterocyclic compounds from the collection of pyrrolyl- and indolylazines were screened using a reporter system. The reporter systems were HeLa uterine cervix carcinoma cells carrying a genetic construct with the luciferase gene under the control of the heat shock proteins gene promoter, HSE. The plasmid was provided by Professor Richard Morimoto (NorthWestern University, USA) [26]. HeLa-luc cells were incubated with substances from the collection at a concentration of 1 μM for 24 h, after which the luciferase activity was determined using a BrightGlo kit (Promega, Southampton, UK) and a Fluorophot Charity multichannel spectrophotometer (Probanauchpribor LLC, St. Petersburg, Russia). The measurement time was 500 ms. One of the most effective compounds was PQ-29 (3-(5-phenyl-1*H*-pyrrol-2-yl)quinoxaline-2(1*H*)-one). This compound was first synthesized in Ural Federal University (UrFU) and prepared according to the published procedure [27].

### 2.2. Cells

C6 rat glioblastoma cells were obtained from the Cell Culture Collection of the Institute of Cytology RAS (St. Petersburg, Russia). Cells were cultured in DMEM/F12 medium (Gibco, Carlsbad, CA, USA) containing 10% fetal bovine serum (FBS; Gibco, Carlsbad, CA, USA), 100 units/mL penicillin, and 0.1 mg/mL streptomycin (BioloT, St. Petersburg, Russia) at 37 °C and 5% CO_2_.

In order to confirm the Hsp70-inducing properties of PQ-29, a reporter plasmid was introduced into C6 glioblastoma cells using the Lipofectamine 3000 transfection reagent (Thermo Fisher Scientific, Waltham, MA, USA). Subsequently, 24 h after transfection, PQ-29 was added to C6-luc cells at a concentration of 0.2, 0.5, and 1 μM over 24 h. The luciferase activity was determined using the BrightGlo kit. As a positive control for the action of chaperone-inducing chemicals, we used heat shock. For this purpose, the cells were incubated at a temperature of 43 °C for 30 min.

### 2.3. Electrophoresis and Immunoblotting

C6 rat glioblastoma cells were incubated with PQ-29 at a concentration of 0.2, 0.5, and 1 μM for 18 h, lysed, and lysates were used for electrophoresis and blotting as described previously [28]. The blot was subsequently incubated with antibodies against Hsp70, clone 3C5 [29], and glyceraldehyde-3-phosphate dehydrogenase (GAPDH, Clone 6C5, Abcam, Cambridge, UK).

To analyze Hsf1 trimers, we applied non-denaturating gradient polyacrylamide gel electrophoresis. The gradient gel was prepared by layering partially blended 4% and 15% solutions from 30% stock of 37:1 acrylamide:bis-acrylamide mixture. Early obtained cell lysates with a concentration of 80 mg were loaded into the gradient gel without adding β-mercaptoethanol, SDS and without heating. Electrophoresis buffer excluded SDS.

### 2.4. RNA Isolation and Real-Time PCR

RNA was isolated using TRIzol (Thermo Fisher Scientific, Waltham, MA, USA) and reverse transcribed using the MMLV RT kit (Evrogen JSC, Moscow, Russia) according to the manufacturer’s instructions. All real-time polymerase chain reactions (RT-PCR) were performed with a CFX96 Real-Time PCR detection system (BioRad, Hercules, CA, USA) using qPCRmix-HS SYBR (Evrogen JSC, Moscow, Russia) according to the manufacturer’s protocol. Amplicon authenticity was confirmed by melt curve analysis. The sequence of primers was as follows for GAPDH: (forward) 5′-ATGATTCTACCCACGGCAAG-3′, (reverse) 5′-CTGGAAGATGGTGATGGGTT-3′; for HSPA1A (Hsp70): (forward) 5′-CAAGAATGCGCTCGAGTCCTA-3′, (reverse) 5′-GGAGATGACCTCCTGGCACTT-3′. GAPDH was used as the normalization control. All primers were obtained from Evrogen JSC (Moscow, Russia). The parameters of the polymerase chain reaction (PCR) were: 5 min of pre-denaturation at 95 °C, followed by 40 cycles of 30 s at 95 °C, 30 s at 65 °C, and 30 s at 70 °C. The data were analyzed for fold change (ΔΔCt) using BioRad CFX software.

### 2.5. Animals

Wistar male rats from Rappolovo animal farm (Russia) weighing 200–250 g on postnatal day 75–80 were used. TBI was performed according to the protocol of Mychasiuk et al. [30] with a minor difference: the height of the drop was 120 cm (instead of 100 cm) [11]. CSF was taken through the large occipital foramen. As a control, CSF from non-injured animals was used. To analyze CSF toxicity, we mixed 50 μL of the CSF sample with 50 μL of cell culture medium and incubated it with C6 cells.

For the analysis of the PQ-29 therapeutical potential on animals, Wistar rats weighing 200–250 g were divided into four groups: not injured (Control, *n* = 10); not injured and treated with PQ-29 (PQ-29, *n* = 9); injured and treated with dimethyl sulfoxide (DMSO) as a vehicle (TBI vehicle, *n* = 10); and injured and treated with PQ-29 (TBI PQ-29, *n* = 9). Therapy was carried out using PQ-29 injections at a rate of 1 mg/kg 3 times a week. The musculoskeletal deficit in the front and hind legs was evaluated by the beam walking test (OpenScience, Russia) on the 30th day after TBI. The testing procedure was recorded on a video with subsequent analysis of the number of slippages of the legs, as described previously [31].

All animal experiments were carried out in accordance with the guidelines for the welfare of animals of the Institute of Cytology, Russian Academy of Science No. F18-00380 (approved on 12 October 2017).

### 2.6. Three Methods Were Utilized to Determine the Physiological Characteristics of C6 Cells Responding to PQ-29

#### 2.6.1. Analysis of Proliferation

Real-time evaluation of C6 cell proliferation was performed using an xCELLigence RTCA DP instrument (ACEA Biosciences, San Diego, CA, USA). To analyze the cell index, C6 cells were introduced into the wells of a 16-well E-plate (10,000 per each well) at the bottom of which a gold electrode was placed. Assessment of the cell index (measurement of cell resistance) was carried out every 10 min. The recording was carried out for three days. The results were analyzed using xCELLigence RTCA DP instrument Software 1.2.

#### 2.6.2. Viability Test

The 3-(4,5-dimethylthiazol-2-yl)-2,5-diphenyltetrazolium bromide (MTT) assay was used to determine the possible toxicity of compound PQ-29. C6 cells were incubated in 96-well plate with the compound PQ-29 at a concentration of 0.1, 0.5, 1.0, 2.0, 5.0, and 10.0 μM for 24 h, after which the level of formazan was determined by a standard method [32]. An amount of 0.5 mg/mL MTT reagent (3-4,5-dimethylthiazol-2-yl-2,5-tetrazolium bromide, Sigma, USA) briefly dissolved in fresh Dulbecco’s modified Eagle’s medium supplemented with F12 (DMEM/F12) was added to each well, replacing the old growth medium. The cells were incubated with MTT for 4 h at 37 °C; after this, the medium containing MTT was removed, and 200 µl of DMSO (Amresco, Solon, OH, USA) was added into each well to dissolve blue formazan crystals in living cells. The optical density was measured on a Fluorofot immunochemistry analyzer system (OOO “PROBANAUCHPRIBOR”, St. Petersburg, Russia) at 570 and 630 nm.

#### 2.6.3. Apoptosis Analysis

Detection of apoptosis was performed using Annexin-V TM 633 (Life Technologies, Eugene, OR, USA) staining. C6 cells were incubated in the presence of CSF from rats for the time indicated in the figures. Then cells were collected, washed in cold phosphate buffer saline (PBS), resuspended in the binding buffer provided by the manufacturer, and stained with Annexin-V Alexa Fluor 647 and propidium iodide (PI) according to the manufacturer’s recommendations. Apoptotic cells were then measured with the aid of the CytoFlex flow cytometer (Beckman Coulter, Miami, FL, USA) using a laser set at 488 (PI fluorescence) and 638 nm (Alexa647 fluorescence) and then analyzed with CytExpert 2.0 (Beckman Coulter, Miami, FL, USA) software.

### 2.7. Immunohistochemistry

At the end of the beam walking test (34 days after TBI), the rats were anesthetized with Zoletil-100 (50 mg/kg, intraperitoneal), perfused with 4% paraformaldehyde, and then decapitated. The brain was extracted and examined by confocal microscopy. Brains from all animals used for immunohistochemical assays were fixed in 4% paraformaldehyde and cryoprotected in 20% sucrose before storage in isopentane at −70 °C. Coronal sections (15 μm) were prepared for morphological and immunohistochemical assay with a Leica CM1510S-1 cryostat (Leica Microsystems, Wetzlar, Germany). The frontal slices were collected at the level of the hippocampus (from bregma −3 mm to −4.3 mm) according to the atlas of Paxinos and Watson [33]. Six alternate series of sections were mounted on SuperFrost Plus slides (Menzel GmbH, Berlin, Germany).

For confocal microscopy, sections were preincubated in blocking solution (2% bovine serum albumin diluted in PBS with 0.1% Tween-20) for 1 h at room temperature, and then processed with Click-iT TUNEL Alexa Fluor 647 kit (Thermo Fisher Scientific, Waltham, MA, USA) according to the manufacturer’s protocol. After rinsing in PBS, the sections were subsequently incubated with 4′,6-diamidino-2-phenylindole (DAPI) for 10 min (1:10,000; Sigma-Aldrich, St. Louis, MO, USA). Fluorescent images were captured by Olympus confocal system FV3000 (Olympus, Tokyo, Japan).

### 2.8. Caspase-3 Assay

The caspase-3 activity assay was performed as described by Denault et al. [34]. Briefly, C6 glioma cells were seeded onto a 12-well plate at a concentration of 2 × 10^5^/mL. After incubation with PQ-29 and/or with CSF samples, cells were harvested by trypsinization and centrifugation at 800× *g* and 4 °C, washed twice in ice-cold PBS, and lysed in a buffer containing 100 mM HEPES pH 7.2, 10% sucrose, 1 mM EDTA pH 8.0, 0.1% CHAPS, and 10 mM DTT. Lysates were subjected to two freeze-thaw cycles at −80 °C and centrifuged at 13,000× *g* for 10 min at +4 °C. The protein concentration in the supernatant was measured with the Bradford assay. Lysate containing a total of 100 µg of protein in 100 µL of lysis buffer was added into the wells of a black 96-well plate (Thermo Fisher Scientific, Waltham, MA, USA) and 20 µM of fluorogenic substrate (DEVD-AMC; Sigma-Aldrich, St. Louis, MO, USA) in 100 µL of lysis buffer was added to each well. The plate was incubated at 37 °C for 2 h, and fluorescent signals were detected using a Varioscan LUX (excitation λ = 355 nm, emission λ = 460 nm; Thermo Fisher Scientific, Waltham, MA, USA). The non-treated cells were used as a control.

### 2.9. Statistics

All data were expressed as the mean ± standard error of the mean (SEM). Statistics were compared using the Mann–Whitney non-parametric test with the aid of GraphPad Prism 8 software. All experiments exclude animal studies were repeated at least three times. A statistical difference was determined by a value of *p* < 0.05.

## 3. Results

### 3.1. Identification of Hsp70 Inducers

**Figure 1:**
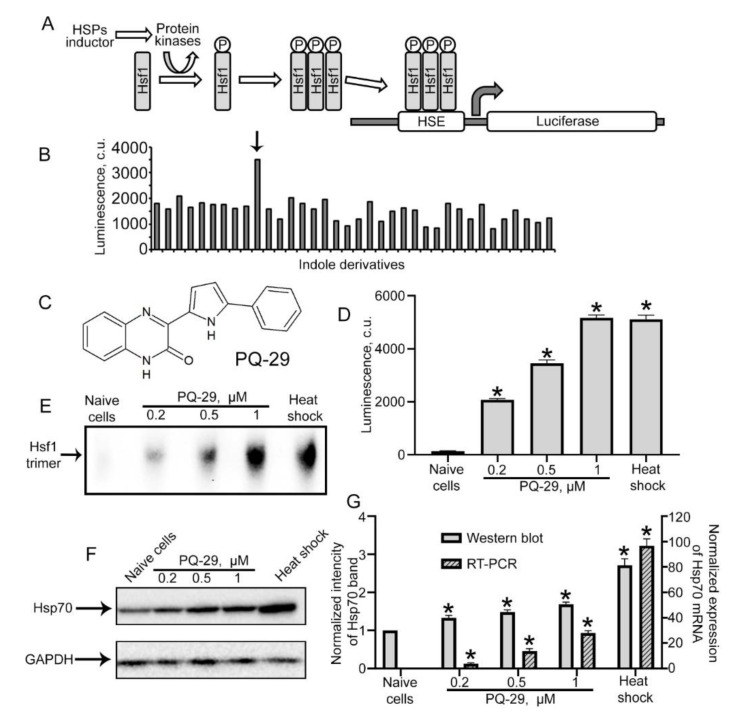
Searching for a new Hsp70 synthesis inducer. (**A**) The general scheme of the test system that we used for screening. (**B**) Typical screening result. The arrow marks the positive compound. (**C**) The chemical formula of the Hsp70 inductor PQ-29. (**D**) The result of the action of PQ-29 on C6-luc cells. Histogram bars illustrate the luciferase activity in cells. (**E**) Data of the Hsf1 trimer analysis. (**F**) Immunoblotting data. C6 cells were treated with PQ-29 in concentrations marked on the figure. Cells were analyzed 18 h after PQ-29 addition. Glyceraldehyde-3-phosphate dehydrogenase (GAPDH) is presented as a loading control. (**G**) The data of real-time polymerase chain reaction (RT-PCR) analysis and the data of immunoblotting quantification are presented. Non-patterned histogram bars show the relative intensity of Hsp70 band from F to intensity of GAPDH band normalized to this meaning for naïve cells accordingly to left *Y*-axis. Patterned histogram bars show the relative amount of mRNA transcribed from the hsp70 gene in C6 cells one hour after PQ-29 addition normalized to the amount of GAPDH mRNA accordingly to right *Y*-axis. Data presented as mean ± standard error of mean (SEM). Statistical significance is indicated as * *p* < 0.05.

The collection from the UrFU was employed to search for the activators of Hsp70 synthesis; the chemicals were selected based on the similarity of their domain to the indole-like pharmacophore found earlier that increased the Hsp70 level [35]. We used the reporter assay comprised of rat C6 glioma cells expressing the luciferase gene under the control of the HSE promotor. This promotor triggers the transcription of multiple genes (including *hsp70*) after interaction with transcription factor Hsf1, which is activated in response to heat shock and other stress inducers (Figure 1A). Measuring HSE-controlled luciferase activity, we found the compound that caused the significant accumulation of luciferase in the cells (Figure 1B). One of the most effective Hsf1 activators was compound PQ-29, whose chemical formula is shown in Figure 1C. According to the reporter assay, PQ-29 activates Hsf1 at a concentration of 1 μM, and the value of luciferase activity is similar to that attained with the principal Hsf1 activator, heat shock (Figure 1D). To check whether PQ-29-mediated activation of heat-shock response employed the common mechanism of Hsf1 trimerization, we performed gel electrophoresis in non-denaturation conditions of C6 cell lysate coupled with Western blotting with the anti-Hsf1 antibody. We found enhanced staining of a band corresponding to the factor trimers (Figure 1E). To further confirm PQ-29-mediated enhancement of *hsp70* gene expression, we measured mRNA content using quantitative PCR and found that one hour after the addition of PQ-29, the mRNA level increased significantly (Figure 1F), though it did not reach the level typical of heat shock. Finally, we analyzed the effect of PQ-29 on the level of intracellular Hsp70 using Western blotting. Incubation of C6 glioma cells in the presence of PQ-29 led to a five-fold increase in the amount of Hsp70 protein in the cells (Figure 1G).

### 3.2. In Vitro Model of Secondary Damage Caused by TBI

**Figure 2:**
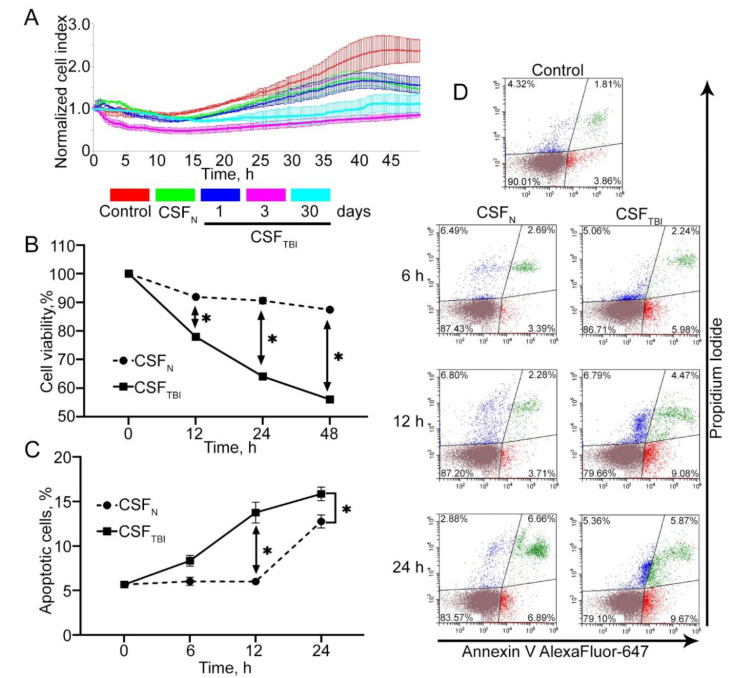
Presentation of an in vitro model for studying secondary damage after traumatic brain injury (TBI). (**A**) Cell index data produced using an xCELLigence device. The graph shows the dynamics of the C6 cellular index placed in a medium containing 30% of rat’s cerebrospinal fluid (CSF), received after 1, 3, or 30 days after TBI (CSF_TBI_). Control: CSF in medium was replaced by phosphate buffer saline (PBS). CSF_N_: CSF from non-traumatized rats was used as a negative control. (**B**) Results of analysis with the aid of the 3-(4,5-dimethylthiazol-2-yl)-2,5-diphenyltetrazolium bromide test (MTT). The CSF sample obtained three days after TBI was incubated with C6 cells for 12, 24, and 48 h. (**C**) The results of Annexin V staining. The measurement was provided with the aid of flow cytometry technique. C6 cells were incubated with CSF for 6, 12, or 24 h. (**D**) The distribution of the cell population according to flow cytometry data. Data of three independent experiments presented as mean ± SEM. Statistical significance is indicated as * *p* < 0.05.

To test the protective power of PQ-29, we employed an in vitro TBI model in which rat C6 glioma cells were treated with the CSF of injured or control rats [11]. First, we found that a concentration of CSF with 50% of total growth medium is the most cytotoxic for C6 cells (Figure S1). Furthermore, we defined the period in which the cytotoxic effect of CSF of injured rats was maximal and performed the experiments in which cells were subjected to CSF taken from animals 1, 3 and 30 days after injury, and the cell index (or more commonly, growth rate) was measured with the aid of xCELLigence technology. It was found that the most significant effect on the cell index was exerted by the CSF obtained three days after the injury; the cell index reduced almost three-fold (Figure 2A). The cytotoxicity of CSF obtained from rats 30 days after injury decreased to the level observed for samples taken three days after injury (Figure 2A). Therefore, in further experiments, we used CSF samples taken three days after the injury. Next, we measured the viability of cells incubated with the CSF of injured rats using the MTT test. These measurements were carried out using an incubation period ranging from 12 h to 48 h. The greatest toxic effect was exerted by CSF from injured rats after an incubation of 48 h with target cells and led to 44 ± 1.3% cell death (Figure 2B). This value contrasts with that obtained for cells incubated with CSF of non-injured rats, proving the relevance of our model of secondary damage.

Finally, we tested the ability of CSF of injured animals to induce apoptosis in rat glioma cells. Analysis of the apoptosis level was performed using Annexin V staining. Because the first signs of apoptosis usually appear earlier than the change in the cell index curve and dehydrogenase activity, the incubation time of CSF with cells before Annexin V staining was reduced to 6, 12, and 24 h. The highest level of apoptosis, 15.54 ± 0.55% of the whole cell population, was recorded after the maximum incubation time (24 h). The most significant difference in apoptosis levels between CSF from naïve and traumatized animals was detected at 12 h of incubation with C6 cells and amounted to 5.99 ± 0.51% (control) against 13.55 ± 0.3% (TBI) (Figure 2C). The distribution of the cell population established by flow cytometry is shown in Figure 2D. The results suggest a high level of traumatized rat toxicity due to the induction of apoptosis, suggesting the appropriacy of the model of secondary damage.

### 3.3. PQ-29 Rescues C6 Cells from the Cytotoxicity of CSF from Traumatized Rats

**Figure 3:**
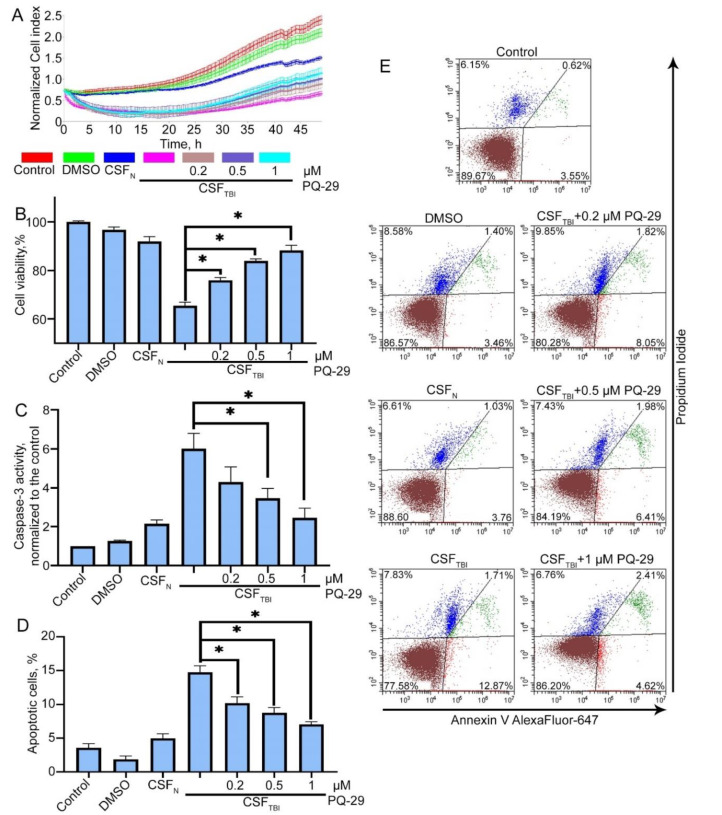
PQ-29 reduces apoptosis and induces the viability of acceptor cells in the presence of CSF from traumatized rats. C6 cells were cultured in the presence of CSF from traumatized rats. CSF:growth medium ratio was 1:1. Control: CSF in medium was replaced by PBS; dimethyl sulfoxide (DMSO) was used as a vehicle; CSF_N_: CSF from non-traumatized rats was used; CSF_TBI_: CSF from traumatized rats was used; PQ-29 was added to cell culture medium immediately after CSF in the concentration marked on the figure. (**A**) Cell index data produced using an xCELLigence device. The graph shows the dynamics of the C6 cellular index placed in a medium containing rat’s CSF. (**B**) The results of the MTT-test. Cells were incubated with CSF for 24 h. (**C**) The results of the caspase-3 activity assay. Cells were incubated with CSF for six hours. (**D**) The results of Annexin V staining. The measurement was provided with the aid of flow cytometry technique. Cells were incubated with CSF for six hours. (**E**) The distribution of the cell population according to flow cytometry data. Data of three independent experiments presented as mean ± SEM. Statistical significance is indicated as * *p* < 0.05.

We assumed that the PQ-29 mediated elevation of Hsp70 content might contribute to enhanced cell protection against the cytotoxicity of CSF from traumatized rats. In these experiments, we used CSF obtained three days after TBI, and Hsp70 production was induced by treatment with 0.2–1 μM PQ-29. First, we evaluated the half maximal inhibitory concentration (IC50) of viability on C6 cells. We estimated that IC50 for PQ-29 was 10.205 μM (Figure S2). Measurement of the cell index showed that PQ-29 at a concentration of 1 μM suppressed the toxic effect of CSF. Accordingly, the cell index increased 1.5-fold, from 0.9 to 1.3, while normal cells without the addition of CSF demonstrated the value of 1.9 (Figure 3A). Data from the MTT assay showed that cell viability elevated from 65.5 ± 1.2% to 83.9 ± 0.83% when using 0.5 μM PQ-29, and up to 88.2 ± 1.52% when using 1 μM PQ-29 (Figure 3B). To validate these data, we visually monitored the state of the cells with a microscope. Corresponding images obtained in transmitted light are presented on Figure S3. There were no visually detectable differences in cell morphology after PQ-29 therapy. Next, we studied the major attribute of apoptosis, caspase-3 activation in cells incubated with toxic CSF. The data of caspase-3 enzymatic assay show that incubation of С6 cells for six hours in the presence of CSF samples from control animals led to the 2.18-fold activation of caspase-3 compared with non-treated cells, incubation of cells in the presence of CSF from injured animals led to the 5.97-fold activation of caspase-3 (compared with non-treated cells). The use of PQ-29 at a concentration of 1 μM reduced the caspase-3 activity to the level of 2.4-fold superiority to non-treated cells (Figure 3C).

The inhibition of apoptosis by PQ-29 was also observed when using Annexin V staining and flow cytometry (Figure 3D,E). After treatment with 0.5 μM of PQ-29, the proportion of apoptotic cells was reduced to 8.39 ± 0.48%, and using 1 μM PQ-29 led to a decrease to 7.03 ± 0.72% (versus 14.58 ± 0.54% without the use of the substance). The distribution of the cell population determined by flow cytometry is shown in Figure 3E.

It is assumed that the Hsp70 inducer PQ-29 should be effective in increasing the viability of cells subjected to the deadly effects of CSF from traumatized rats.

### 3.4. Therapeutic Effect of PQ-29 in a Rat Model of TBI

**Figure 4:**
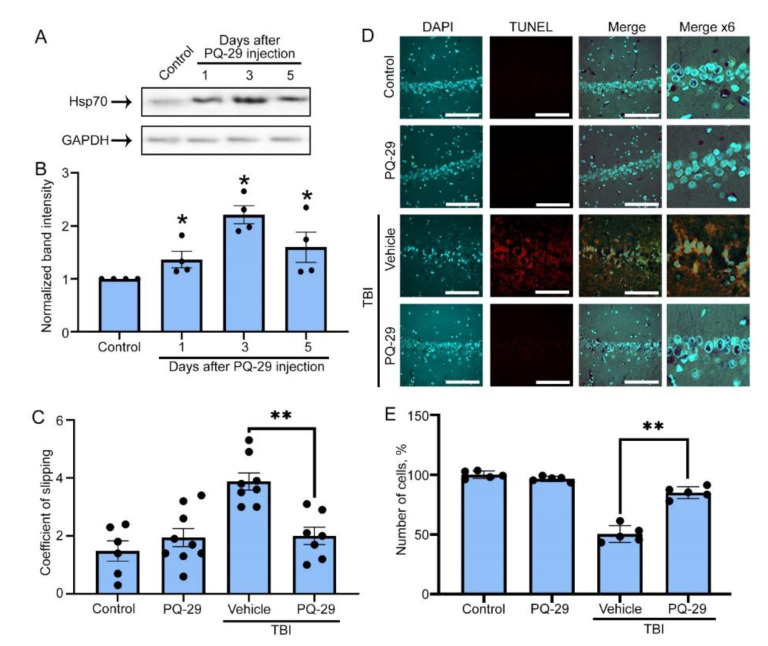
PQ-29 reduces post-traumatic neurodegeneration and saves motor function of traumatized rats. (**A**) The result of rat’s brain immunoblotting analysis. Brain lysates were stained with anti-Hsp70 and anti-GAPDH antibodies on one, three or five days after an intraperitoneal injection of PQ-29. (**B**) The result of band intensity quantification from section A is presented. The histogram bars show the average normalized band intensity of Hsp70 quantified based on three independent experiments. (**C**) Rats were anesthetized and divided into four groups: TBI groups were traumatized, the other two groups were not. The PQ-29 groups were treated with intraperitoneal PQ-29 injections three times a week. DMSO was used as a vehicle. The beam walking test was employed to define the changes in locomotor function, as described in the text. The coefficient of slipping defines the height of the histogram bars. (**D**) The confocal microscopy data are presented. Rats’ brains from different groups, as described in section A, were sliced and stained with 4′,6-diamidino-2-phenylindole (DAPI) (cyan) and Click-It TUNEL kit (red). The CA1 hippocampus field is shown. Scale bar 10 µm. (**E**) The number of cell nuclei stained with DAPI was normalized to that in hippocampus of control animals and presented as histogram bars. For each group, no less than 500 cells were counted. Data presented as mean ± SEM. Statistical significance is indicated as * *p* < 0.05 and ** *p* < 0.01.

Next, we examined the therapeutic potential of PQ-29 in the rat TBI model. Firstly, we proved that a PQ-29 intraperitoneal injection increased Hsp70 level in the rat’s brain. Using Western blotting, we analyzed the Hsp70 content in samples of brain tissue at one, three and five days after injection of the compound (Figure 4A). According to Western blot data, a two-fold increase in Hsp70 band intensity occurred on the third day. A high level of chaperone persisted until the fifth day, when it exceeded 1.6 times the normal level (Figure 4B). To analyze the therapeutic properties of PQ-29, rats were divided into four groups of 10 animals each (control, treated with PQ-29, traumatized and treated with DMSO as a vehicle, traumatized and treated with PQ-29). During the 30 days after the injury, the animals received intraperitoneal injections of PQ-29 (1mg/kg of body weight) three times a week. After this, the motor function of the animals was assessed using the beam walking test, and the level of neuronal death was assessed using histochemical staining of transverse sections of the brain with DAPI and using the TUNEL kit in the area of the hippocampus CA1 field. This region was chosen because of the compact arrangement of neurons, and also because the hippocampus forms the wall of the lateral ventricles and, accordingly, we assumed that it is primarily exposed to toxic factors contained in the CSF.

The graph in Figure 4С shows the absolute values of the coefficient of slipping obtained from the taper track test. Increased values corresponded to impaired motor function. One month after TBI in rats, this parameter was 4.6 ± 0.77; at the same time, in rats treated with PQ-29, the coefficient of slipping was lower, 2.27 ± 0.48, which was not statistically distinct from the two control groups (1.97 ± 0.57 and 2.17 ± 0.41) (Figure 4C). Immunohistochemical staining showed a significant reduction in the number of neurons in the CA1 field of the hippocampus of injured rats, down to 48.3 ± 9.6% as compared to non-injured animals (Figure 4B,C). The therapy partially prevented neuronal death, elevating the proportion of surviving cells to 87.54 ± 6.4% related to that of the control. The results obtained when the brain sections were stained with the TUNEL kit showed that the majority of neurons were stained, whereas the administration of PQ-29 completely blocked apoptosis and stopped neurodegeneration (Figure 4D,E). Thus, the novel Hsp70 inducer demonstrated its effectiveness as a promising tool for rehabilitation therapy after traumatic injury.

### 3.5. PQ-29 Reduces the Cytotoxicity of CSF from Traumatized Rats

**Figure 5:**
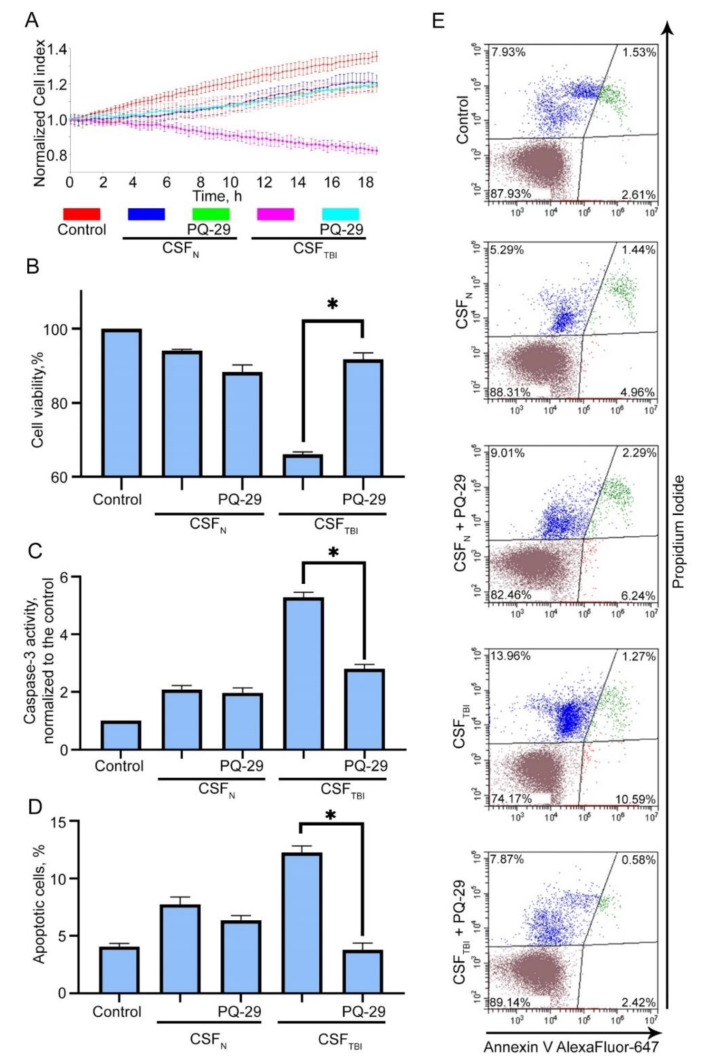
The treatment of traumatized animals with PQ-29 reduced the cytotoxicity of rat’s CSF. CSF samples were collected from traumatized and treated rats 34 days after TBI, mixed with growth medium (in volume ratio 3:7), incubated with C6 cells, and analyzed as described above. Control: CSF replaced by PBS; CSF_N_: growth medium containing CSF from non-traumatized rats; CSF_TBI_: growth medium containing CSF from traumatized rats; PQ-29: growth medium containing CSF from rats treated with PQ-29. (**A**) Cell index data produce using an xCELLigence device. The graph shows the dynamics of the C6 cellular index placed in a medium containing 30% of rat’s CSF. (**B**) The results of the MTT-test are presented. Cells were incubated with CSF for 24 h. (**C**) The results of the caspase-3 activity assay were demonstrated. Cells were incubated with CSF for six hours. (**D**) The results of Annexin V staining are presented. The measurement was provided with the aid of flow cytometry technique. Cells were incubated with CSF for six hours. (**E**) The distribution of the cell population according to flow cytometry data is presented. Data of three independent experiments presented as mean ± SEM. Statistical significance is indicated as * *p* < 0.05.

In order to further analyze the protective effect of PQ-29 in inhibiting the mechanisms of secondary damage, we compared the cytotoxicity of CSFs from injured rats and those who were traumatized and received PQ-29. In these experiments, CSF obtained one month after TBI in rats from different groups (see the previous section) were added to C6 rat glioma cell cultures and analyzed using xCELLigence, the MTT test, and Annexin V staining. We found that the cell index after treatment with CSF obtained one month after injury decreased by approximately 40% (from 1.3 to 0.8), while the CSF of injured rats treated with PQ-29 reduced the cell index by only 20–25%, down to 1–1.05% (Figure 5A). The results of the MTT assay confirmed the efficiency of PQ-29 in reducing the cytotoxic potential of CSF of injured animals. The CSF of injured untreated rats reduced the number of acceptor cells to 66.2 ± 1.9%, and the CSF of treated animals did not cause significant cell death and accounted for 94 ± 5.5% of the surviving cells, while the number of surviving cells after treating the CSF of non-injured rats was 91.6 ± 1.1% (Figure 5B). Then we tested the caspase-3 activity in acceptor cells incubated with CSF from experimental rats. We showed that treatment of traumatized rats with PQ-29 led to reducing caspase-3 activation from 5.31 ± 0.46-fold to 1.81 ± 0.22-fold compared to non-treated cells% (Figure 5C). The apoptosis level induced by the CSF of injured animals was 11.86 ± 0.57% (measured with the aid of Annexin V staining), treatment with PQ-29 reduced the proportion of apoptotic cells to 3 ± 0.3%, which practically corresponds to the apoptosis level of 6.4 ± 0.36% caused by the CSF from non-injured rats (Figure 5D). Such results prove that PQ-29 not only increases the survival of acceptor cells but also reduces the cytotoxic potential of CSF.

## 4. Discussion

TBI leads to significant and massive lesions of brain tissue. The treatment strategy used in clinics focuses on blocking bleeding [36] and controlling intracranial pressure to relieve the acute phase of the pathological condition. Nevertheless, the process of neurodegeneration after head injury (secondary damage) can continue for weeks and months. Ultimately, the development of such pathological processes can lead to impaired motor function, memory deficit, and the formation of anxiety-like behavior in patients. Glutamate receptor antagonists, anti-inflammatory agents, neuroprotectors, and others can be used as therapeutic agents to block secondary damage processes associated with the long-lasting, gradual apoptosis processes [37].

In animal TBI models, depending on the amplitude of the traumatic effect, the peak of apoptosis can be observed after 48 h or even after two weeks [38]. Correspondingly, the moiety of potentially harmful factors can accumulate in CSF, including glutamate, reactive oxygen species, nitric oxide [39,40], and protein aggregates [11,41], that together create a cytotoxic environment for adjacent brain cells [42].

This notion emphasizes the relevance of CSF-target cell models in the study of this secondary damage phenomenon; we proved this in experiments when the cells treated with CSF of injured animals demonstrated a pattern of neurodegeneration, apoptosis, cell death and others (Figure 2).

Using this model, we tested a novel Hsp70 inducer capable of increasing the chaperone-mediated cytoprotection in brain cells. This molecule contained an indole group similar to that found by Lin and co-authors [35]. The new compound, PQ-29, was found to activate Hsf1-controlled transcription by converting the latter molecule in trimeric form, as was shown for most of the activators. PQ-29 elevated the synthesis of mRNA and protein accumulation in various mammalian cells (data not shown). More importantly, this compound was shown to elevate the Hsp70 amount in brain tissues, and its level was kept constant for three days (Figure 4A,B). The other beneficial property is its safety. The IC50 of PQ-29 for viability of C6 cells was found to be 10.2 µM (Figure S2), which is twice as large as that of U-133, another Hsp70 inducer shown earlier to protect rat dopaminergic neurons from Parkinson’s disease pathogenesis [18,43].

A significant advantage of PQ-29 over analogs is that its administration can reduce caspase-3 activity (Figure 3C and Figure 5C). At the same time, one of the most promising inducers, celastrol, causes the accumulation of caspase-3 and activates apoptosis [44]. Another chaperone-inducing compound is geranyl-geranyl-acetone [45], which was also tested in a TBI model. However, its further clinical application may be hampered by the extremely high doses employed in practice [46]; a single injection was 800 mg/kg, which is 800-fold more than the effective dose of PQ-29 (Figure 4).

Importantly, PQ-29 demonstrated abilities to increase cell viability in the presence of CSF (Figure 3A,B,D,E) and to reduce the cytotoxicity of CSF derived from the injured and treated with the inducer animals (Figure 5A,B,D,E). Of note, in the latter case, not only the level of apoptosis decreased, but also necrosis. This may likely be due to the versatility of the cytoprotective effect of Hsp70 induced by PQ-29. In addition to anti-apoptotic activity, Hsp70 can reduce the number of cytotoxic protein complexes that form in brain cells after injury and can accumulate in the CSF [11]. We believe that it was the decrease in the number of protein aggregates in the CSF of injured animals after PQ-29 treatment that caused the reduction in the necrosis of acceptor cells (Figure 5E).

One of the major problems of neurodegenerative disease therapy is a difficulty with the diagnosis of the pathological process [47], which significantly complicates therapeutic efficacy [48]. Rehabilitation therapy for TBI is almost always performed after the injury, i.e., when the administration of Hsp70 inducer can really be effective. This may be validated by our results, showing that the activation of the chaperone system should be extremely effective due to the clear understanding of the moment when the neurodegenerative process begins and the ability to immediately begin treatment. We successfully confirmed this hypothesis in the rat TBI model. The treatment of injured animals with PQ-29 led to a decrease in apoptosis in the hippocampal CA1 neurons, prevented the development of motor disorders, and reduced the cytotoxic activity of CSF from traumatized animals. As secondary damage after TBI is a multi-factor problem, we suggest that combined therapy is necessary which may include Hsp70 inducers, aggregates formation inhibitors, and antioxidants.

## 5. Conclusions

Summing up the work undertaken in this study, it should be noted that we screened compounds from the collection of pyrrolyl- and indolylazines, discovered a new inducer of Hsp70 synthesis, established the mechanism of action of the inducer, and proved the protective effect of the compound on cellular and animal models of the post-traumatic state. According to our results, the efficacy of PQ-29 as a therapy factor used after TBI is due to an accumulation of Hsp70 in brain cells, causing a reduction in caspase-3 activity, and the suppression of apoptosis, which—at least partially—contributes to the prevention of neurodegeneration.

## Supplementary Materials

The following are available online at https://www.mdpi.com/1999-4923/12/5/414/s1, Figure S1: Determination of the cytotoxic effect of cerebrospinal fluid control (CSF-N) and injured (CSF-TBI) rats, Figure S2: Determination of half maximal inhibitory concentration (IC50) of PQ-29 for viability of rat C6 glioma cells, Figure S3: General view in transmitted light of C6 cells after culturing them for 24 hours in the presence of CSF control rats (CSF_N_), injured rats (CSF_TBI_) and in the presence of PQ-29 at the concentrations indicated in the figure.
