# Supplementary material for: Pyrrolylquinoxaline-2-One Derivative as a Potent Therapeutic Factor for Brain Trauma Rehabilitation
Source: Pharmaceutics. 2020 Apr 30;12(5):414. doi: 10.3390/pharmaceutics12050414 (PMC7285016; doi:10.3390/pharmaceutics12050414)
Supplement: Supplementary file 1 [file pharmaceutics-12-00414-s001.pdf]

pharmaceutics

Supplementary Materials: Pyrrolylquinoxaline-2-One Derivative as a Potent Therapeutic Factor for Brain Trauma Rehabilitation

Elizaveta A. Dutysheva, Marina A. Mikeladze, Maria A. Trestsova, Nikolay D. Aksenov, Irina A. Utepova, Elena R. Mikhaylova, Roman V. Suezov, Valery N. Charushin, Oleg N. Chupakhin, Irina V. Guzhova, Boris A. Margulis and Vladimir F. Lazarev

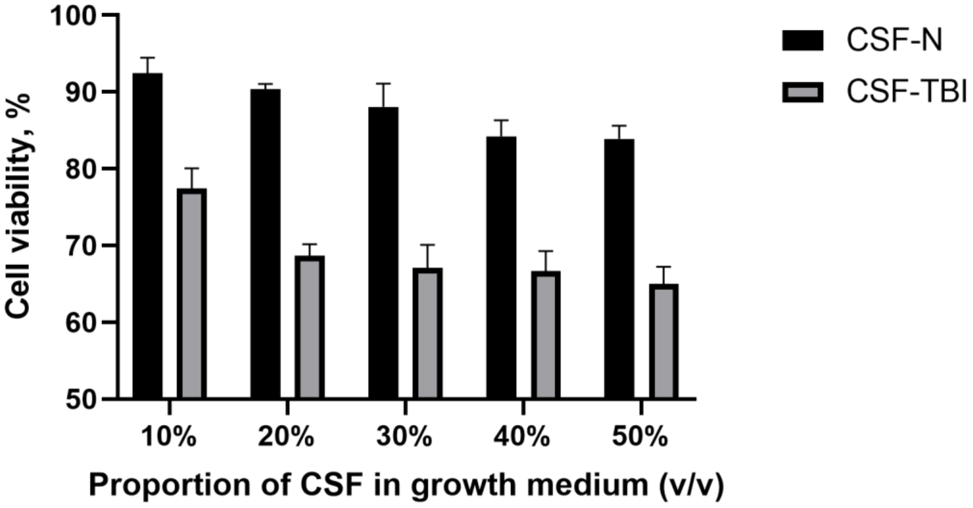

Figure S1. Determination of the cytotoxic effect of cerebrospinal fluid control (CSF-N) and injured (CSF-TBI) rats. The data obtained using the MMT test are presented. C6 cells were cultivated for 24 hours in the presence of different concentration of CSF as signed on figure.

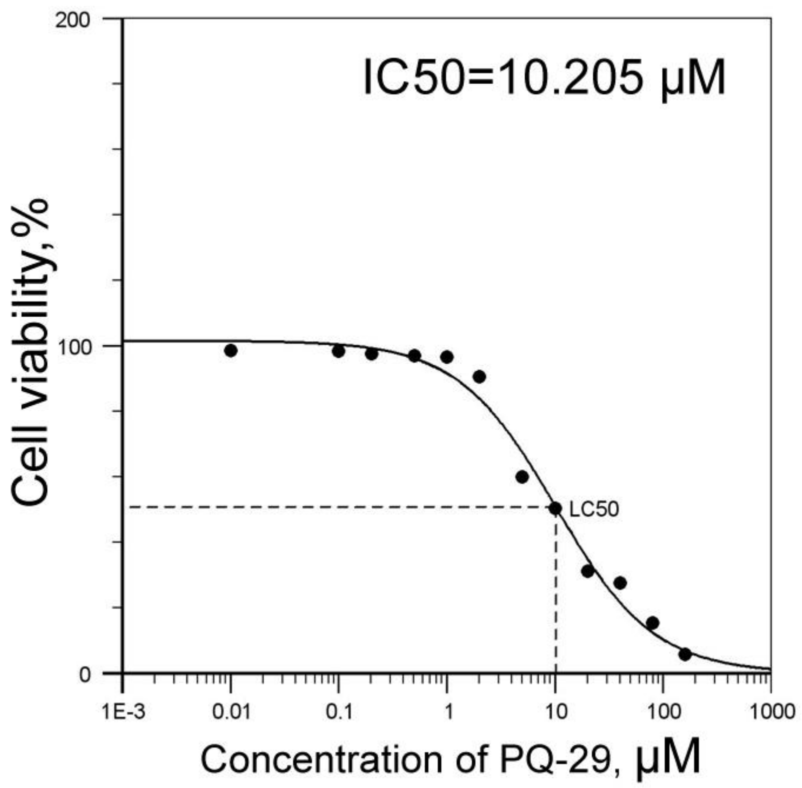

Figure S2. Determination of half maximal inhibitory concentration (IC50) of PQ-29 for viability of rat C6 glioma cells. Cell death rate was determined using a CytoTox96 kit. (Promega, UK).

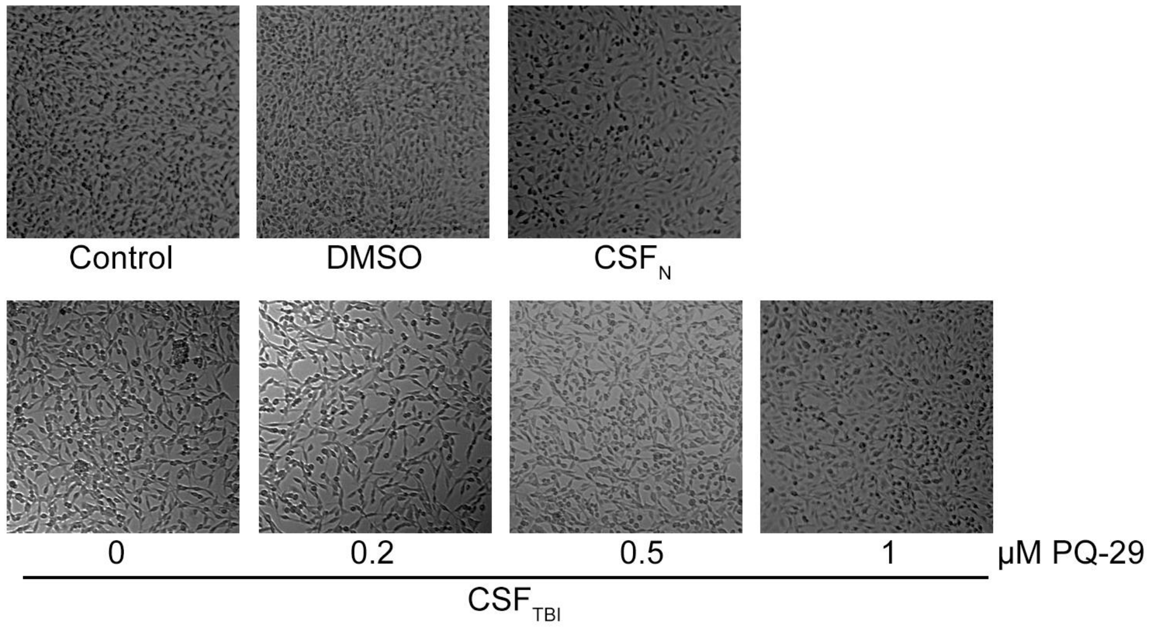

Figure S3. General view in transmitted light of C6 cells after culturing them for 24 hours in the presence of CSF control rats (CSF_N), injured rats (CSF_{TBI}) and in the presence of PQ-29 at the concentrations indicated in the figure.
